# Development of microorganisms for cellulose-biofuel consolidated bioprocessings: metabolic engineers’ tricks

**DOI:** 10.5936/csbj.201210007

**Published:** 2012-11-08

**Authors:** Roberto Mazzoli

**Affiliations:** a Department of Life Sciences and Systems Biology, University of Torino, Via Accademia Albertina 13, 10123 Torino, Italy

**Keywords:** metabolic engineering, butanol, ethanol, hydrogen, cellulosome, cellulase

## Abstract

Cellulose waste biomass is the most abundant and attractive substrate for “biorefinery strategies” that are aimed to produce high-value products (e.g. solvents, fuels, building blocks) by economically and environmentally sustainable fermentation processes. However, cellulose is highly recalcitrant to biodegradation and its conversion by biotechnological strategies currently requires economically inefficient multistep industrial processes. The need for dedicated cellulase production continues to be a major constraint to cost-effective processing of cellulosic biomass.

Research efforts have been aimed at developing recombinant microorganisms with suitable characteristics for single step biomass fermentation (consolidated bioprocessing, CBP). Two paradigms have been applied for such, so far unsuccessful, attempts: a) “native cellulolytic strategies”, aimed at conferring high-value product properties to natural cellulolytic microorganisms; b) “recombinant cellulolytic strategies”, aimed to confer cellulolytic ability to microorganisms exhibiting high product yields and titers.

By starting from the description of natural enzyme systems for plant biomass degradation and natural metabolic pathways for some of the most valuable product (i.e. butanol, ethanol, and hydrogen) biosynthesis, this review describes state-of-the-art bottlenecks and solutions for the development of recombinant microbial strains for cellulosic biofuel CBP by metabolic engineering. Complexed cellulases (i.e. cellulosomes) benefit from stronger proximity effects and show enhanced synergy on insoluble substrates (i.e. crystalline cellulose) with respect to free enzymes. For this reason, special attention was held on strategies involving cellulosome/designer cellulosome-bearing recombinant microorganisms.

## Construction of microorganisms for consolidated bioprocessings: economic motivation and strategies

Cellulose biomass is the largest waste produced by human activities and the most attractive substrate for “biorefinery strategies” to produce high-value products (e.g. fuels, plastics, enzymes) [1, 2]. The current price for cellulosic biomass, i.e. $50/ metric ton, makes it less expensive than all other energy sources [1]. Nonetheless, plant biomass is highly recalcitrant to biodegradation. No natural microorganisms able of efficient single-step cellulosic biomass fermentation, i.e. consolidated bioprocessings (CBP), into valuable products have been isolated so far. Traditional biomass bioconversion processes require extensive feedstock pre-treatment (e.g. by steam-explosion and/or acid treatment) and the addition of exogenously produced cellulases [3, 4]. From study to study, depending on calculation methods and base-case to be compared, enzyme production impact on the whole bioconversion process cost has been widely differently estimated [5, 6]. However, the cost of added enzyme does not show a decreasing trend over time and still is a major constraint to cost-effective processing of cellulosic biomass [6]. At the low end of recent estimates, i.e. 0.50 $ per gallon ethanol, the cost of added enzymes is comparable to feedstock purchase cost thus eliminating the economic advantage of cellulosic biomass relative to corn [1, 6]. Process cost reduction can be obtained by CBP through simpler feedstock processing, lower energy inputs, higher rates and yields, although, in some cases, economic advantages of CBP might have been over-estimated or evaluated on assumptions that may be difficult to realize [4–7].

**Figure 1:**
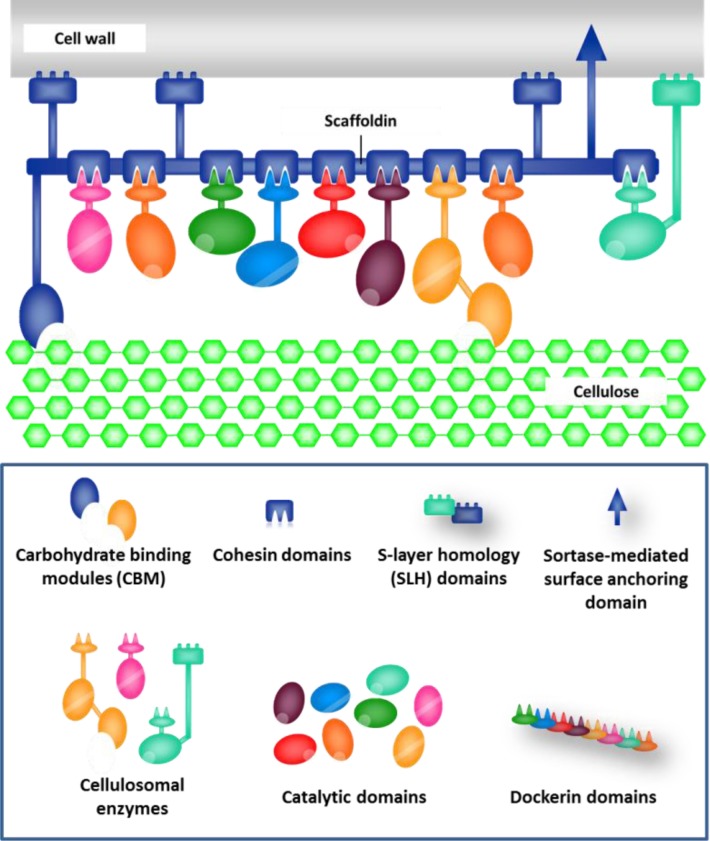
**Simplistic model of a cellulosome that includes only one anchoring scaffoldin**. The scaffolding protein (blue) binds the enzymatic components through cohesin-dockerin interactions, enhances the cellulosome affinity for cellulose through the CBMs, and anchors the cellulosome complex to the cell surface through either non-covalent (by means of multiple S-layer homology domains) or covalent (mediated by sortases) bonds. Apart from the catalytic domains, cellulosomal enzymes include dockerin modules and, possibly, additional domains (*e.g*. CBM, SLH) (modified from [60]).

Native plant degrading microorganisms synthesize extracellular multiple enzyme systems that have different substrate specificities (e.g. cellulases, xylanases, pectinases) and catalytic mechanisms (i.e. endoglucanases, exoglucanases, processive endoglucanases and β-glucosidases) [3, 8, 9]. Enzymatic proteins can be either free or physically associated to form complexes called “cellulosomes” [3, 8]. Cellulosomes are typical of anaerobic strains (e.g. *Clostridium spp*. and *Ruminococcus spp*.) and are by far the most efficient biochemical systems for cellulose degradation [3, 8, 10–12]. Cellulosome architecture is organized by “scaffoldins”, which are able to recruit catalytic proteins by cohesin-dockerin interactions and improve complex affinity for the substrate via carbohydrate binding domains (CBMs) [3, 8] (Figure 1). In some strains, e.g. *Clostridium thermocellum*, *Clostridium cellulovorans* and *Ruminococcus flavefaciens*, scaffoldins also provide cell wall binding through covalent or non-covalent interactions [8].

Construction of recombinant microorganisms for CBP can be pursued via two alternative approaches [2–4]. Native cellulolytic strategies (NCSs) aim at introducing and/or improving high-value product biosynthetic pathways into natural cellulolytic strains. The purpose of recombinant cellulolytic strategies (RCSs) is to confer cellulolytic ability to microorganisms with valuable product formation properties and include heterologous cellulase expression. The next sections will describe limitations of each of these approaches and cutting edge solutions that were applied or could be employed in the construction of recombinant cellulolytic strains.

## Strain development via native cellulolytic strategies

NCSs are currently hampered by some common limitations. Most native cellulolytic strains have been recently isolated from natural environments and are poorly characterized. Genetic manipulation tools have been set-up for relatively few of them. As far as cellulolytic fungi are concerned, most engineering efforts have been addressed to increasing cellulase production, although there is increasing interest in biofuel production engineering [6, 13–14]. *C. thermocellum* and *C. cellulolyticum* are the most established cellulosome-forming microorganisms [6, 15–17]. Furthermore, the metabolism of few strains has been investigated in depth, with *C. cellulolyticum* as by far the best characterized microorganism [7, 18]. Even in strains with fully sequenced genomes, many genes are annotated as hypothetical while others may have been improperly annotated since their function was deduced on the base of amino acid sequence homology only [7].

In addition, problems connected with the construction of recombinant strains for specific compound production occur. Biomass biorefinery potential for sustainable production of a large spectrum of high value products, such as building block chemicals (e.g. succinic acid, lactic acid, isoprene), higher alcohols, lipidic compounds, fine chemicals (e.g. vitamins, antibiotics), has been extensively reviewed [19–21]. Production of H_2_, ethanol and butanol has been targeted in this study because of their huge potential as biofuels [1, 2, 7].

### Improving hydrogen production in cellulolytic microrganisms

A number of anaerobic cellulolytic bacteria, including several Clostridia (e.g. *C. cellulolyticum*, *C. cellulovorans*, *C. termitidis* and *C. thermocellum*), Ruminococci (*e.g*. *R. albus*) and the extreme thermophile *Caldicellulosiruptor saccharolyticus*, are able to produce H_2_ [7, 22]. However, H_2_ yields obtained by direct cellulose fermentation are usually low because of other metabolic pathways (e.g. producing acids, alcohols and ketones) which compete with proton reduction to H_2_ for electron donors, i.e. reduced Ferredoxin (Fd_red_) and/or NAD(P)H [7, 23]. H_2_ yields of mesophilic cellulolytic bacteria generally range from 1 to 2 mol H_2_/mole hexose sugar, while values close to the theoretical maximum of 4 mol H_2_/ mole hexose sugar can be obtained by hyper thermophiles such as *C. saccharolyticus* [7, 23].

**Figure 2:**
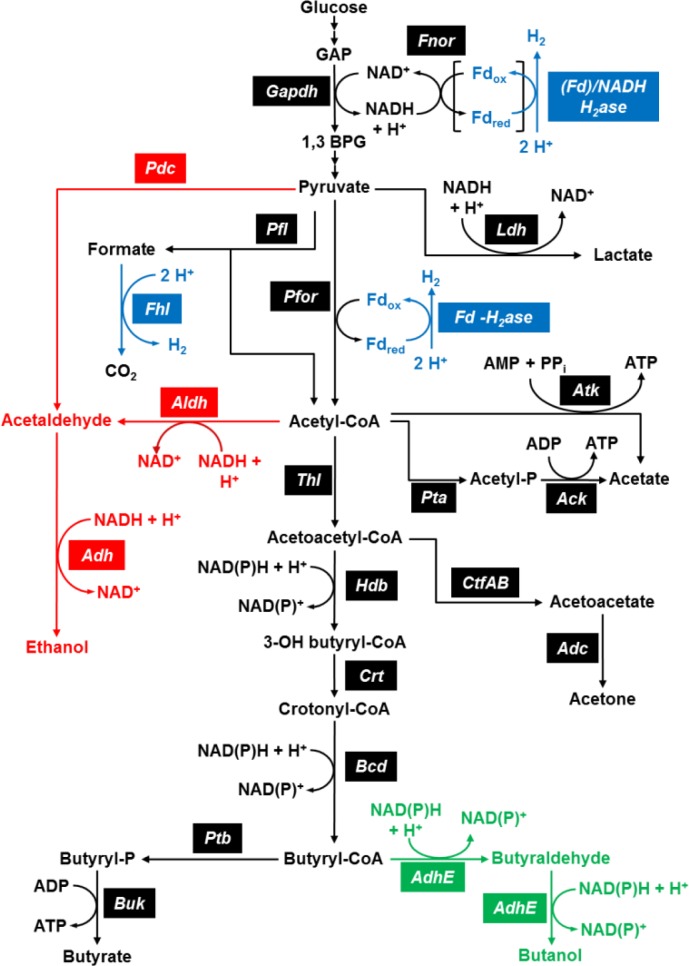
**Overview of** ***Clostridium sp**.* **central metabolic pathways**. Pathways for butanol, ethanol and hydrogen production are highlighted in green, red and blue, respectively. Redox reactions involving NAD(P) or Fd and ATP generating reactions have been indicated. Glucose is catabolized through the Embden-Meyerhof route. Formate oxidation to H2 and CO2 by Formate Hydrogen Lyase (Fhl) occurs in enteric bacteria and in some species of *Clostridium*, although has not been observed in cellulolytic species like *C. thermocellum* so far [7]. Abbreviations: 1,3 BPG, 1,3 bisphosphoglycerate; Acetyl-P, acetyl phosphate; Butyryl-P, butyryl phosphate; Fd, ferredoxin; Ack, acetate kinase; Adc, acetoacetate decarboxylase; AdhE, aldehyde/alcohol dehydrogenase; Atk, acetate thiotransferase; Bcd, butyryl-CoA dehydrogenase complex; Buk, butyrate kinase; Crt, crotonase; CtfAB, acetoacetyl-CoA:acyl-CoA transferase; Fnor, ferredoxin:NAD(P)+ oxidoreductase; H2ase, hydrogenase; Hbd, 3-hydroxybutyryl-CoA dehydrogenase; Ldh, lactate dehydrogenase; Pfor, pyruvate:ferredoxin oxidoreductase; Pdc, pyruvate decarboxylase; Pta, phosphotransacetylase; Ptb, phosphotransbutyrylase; Thl, thiolase.

Fermentative pathways either promoting or competing with H_2_ biosynthesis have mostly been studied in *Clostridium sp*. [7, 24] and are depicted in Figure 2.

H_2_ases are regarded as the most efficient enzymes catalyzing either proton reduction (i.e. H_2_-evolving H_2_ases) or H_2_ oxidation (i.e. “uptake” H_2_ases) [24]. Construction of strains with improved H_2_ production has been carried out by deletion of genes encoding “uptake” H_2_ases and/or overexpression of H_2_-evolving enzymes [25–26]. Engineering more efficient H_2_-evolving proteins has also been proposed to increase H_2_ yield and/or productivity [27–28]. However, these strategies appear to have limited potential since all H_2_ases, although preferentially directed, are known to be reversible [7, 29].

Greater H_2_ yields could be obtained through repression of competing pathways (i.e. producing more reduced end-products than acetate) (Figure 2) [7, 29]. Deletion of *ldhA* in hemicellulolytic *Thermoanaerobacterium sp*. either had no effect or increased H_2_ production by 2-fold, depending on the strain, while repression of acetate production by *ack* and *pta* knockout reduced H_2_ yields by more than 25 fold [30–31]. Recent papers reported repression of lactate and/or acetate production in *C. cellulolyticum* and *C. thermocellum* by Ldh or Ack/Pta gene inactivation, respectively, but effects on H_2_ production were not studied [16, 32–34]. Suppression of butyrate production was recently obtained in *C*. *acetobutylicum* and *Clostridium butyricum* by inactivation of *hdb* gene encoding 3-hydroxybutyryl-CoA dehydrogenase [35–36]. However, H_2_ production strongly decreased in both strains. Elimination of ethanol formation alone did not increase H_2_ production in *C. butyricum* [37].

Actually, the H_2_ metabolic network in strict anaerobes is very complicated. A paradigm for this is the high diversity of clostridial hydrogenases and the existence of multiple forms, likely involved in different functions (e.g. redox balancing, derivation of energy from H_2_ oxidation, proton respiration and/or proton-gradient build-up) within one species [24]. Recently, members of the so called bifurcating hydrogenases have been identified in several clostridia, including cellulolytic strains [24, 38]. Among them, butyryl-CoA dehydrogenases/EtfAB (Bcd/EtfAB) complex from *Clostridium kluyveri* couples NADH-dependent exergonic reduction of crotonyl-CoA to butyryl-CoA to endergonic reduction of Fd which can be used for H_2_ production [38]. Discovery of such complex in clostridia provides a clue to H_2_ yield decrease in strains in which butyrate production was suppressed by Hdb inactivation. More detailed understanding of the metabolic networks involved in H_2_ production is definitely essential for successful engineering of H_2_ hyper producing strains.

It is worth to note that, even if 4 mol H_2_/mole glucose yield was attained, still H_2_ production from cellulose would not be competitive with other cellulosic biofuels because of lower yields and higher waste by-product disposal [27–28]. Theoretical analyses have suggested that yields close to the stoichiometry maximum of 12 moles H_2_/mole glucose are possible by redirecting glucose catabolism through the pentose phosphate pathway or by further acetyl-CoA oxidation through citric acid cycle [27–28]. More profound metabolic modification of natural cellulolytic strains will be necessary to assess *in vivo* feasibility of such strategies.

## Challenges for cellulosic ethanol/butanol production by CBP

Metabolic engineering strategies aimed at developing ethanol and/or butanol hyperproducing strains face two main challenges: a) the construction of solvent tolerant strains; b) the achievement of high solvent yield, titer and productivity.

Distinct biochemical systems are generally involved in solvent resistance and biosynthesis. “Titer gap” is defined as the difference between the maximum concentration of a compound that is tolerated when it is added to a culture and the maximum concentration of that compound that is biosynthesized by a strain [6]. The development of *C. thermocellum* strains able to tolerate added ethanol concentrations exceeding 50 g/l has been reported. However, the maximum ethanol titer produced by this organism remains at about 25 g/l [6]. A number of engineered strains, such as *Termoanaerobacterium saccharolyticum*, showed solvent production titers exceeding the same solvent tolerance exhibited in exogenous addition experiments [6, 39]. The latter observations provide increasing support that with sufficient effort, stoichiometric yields of engineered products can be achieved.

### Development of solvent tolerant strains

The main solvent toxicity is attributed to chaotropic effects on biological membranes [40–41]. Compound toxicity is related to its partition in an equimolar mixture of octanol and water, i.e. log P [40]. The higher is solvent polarity the lower is log P. Molecules with log P below 1 or above 4 are scarcely toxic since they are too hydrophilic to enter the membranes or too hydrophobic and therefore not bioavailable, respectively [40]. In this respect, n-butanol (log P 1) is more toxic than ethanol (log P = -0,18). Even in native solvent producers, such as *C. acetobutylicum*, 50% growth inhibition occur for butanol concentration as high as 7–13 g/l and metabolism ceases once solvent reaches 20 g/l [41–42]. Continuous extraction of solvents form the culture medium or two-phase (organic-aqueous) fermentation systems can be employed to overcome solvent toxicity, but they increase industrial process complexity and/or cost [39–40, 42]. The development of strains with superior tolerance features is therefore essential for sustainable production of biofuels [41].

Solvent accumulation within biological membranes increases membrane fluidity and negatively affects membrane processes, e.g. energy generation and nutrient transport [40]. Moreover, solvents may cause protein and RNA unfolding and degradation and DNA and lipid damage [40–41]. In this respect, proteins involved in cellulose hydrolysis are less affected than cells by high solvent concentration and cellulosomes appear less sensitive than free cellulases [43–44]. In response, cells induce complex stress mechanisms that include alterations in cell envelope composition, biosynthesis of heat-shock proteins and solvent active transporters (*i.e*. efflux pumps) and changes in cell size and shape [40–41]. However, the activation of solvent resistance systems increases cell energy expenditure. High energy costs are associated with efflux pumps and repair or re-synthesis of damaged macromolecules [40–41]. The consequences of such system activation on cell energy balance should be included in theoretical calculations of maximum solvent production yields.

Development of solvent tolerant strains has been performed by different strategies. In some cases solvent tolerant mutants also showed increased solvent production. Utilization of random mutagenesis by chemical or physical methods or by using transposable genetic elements has been reported [41]. *C. beijerinckii* BA101, a butanol-tolerant mutant obtained by chemical methods, showed cell inhibition at 23 g/l butanol rather than 11 g/l typical of the wild-type (WT) strain as well as improved solvent production [42]. An alternative strategy relies on overexpression of proteins involved in solvent resistance (e.g. heat-shock proteins, efflux pumps, enzymes changing membrane lipid composition) [45–47]. The overexpression of GroES and GroEL in *C. acetobutylicum* resulted in 85% less growth inhibition by butanol and 30% improved butanol production [45]. Global approaches, e.g. the construction of genomic and deletion libraries and the utilization transcriptomic and proteomic techniques, have been employed so as to expand the number of genes identified as involved in solvent tolerance [41]. The construction of a *C. acetobutylicum* genomic library led to the identification of 16 genes contributing to butanol tolerance [48]. The overexpression of one of them, i.e. CAC1869, in *C. acetobutylicum* resulted in 81% increase in cell density in a butanol-challenged cultures. A more straightforward approach to achieve solvent tolerant phenotype is *in vivo* directed evolution under selective pressure. Cellulolytic *C. thermocellum* strains able to tolerate ethanol concentrations as high as 80 g/l were developed by adaptation approaches [49–50]. Whole genome shuffling (WGS) can be used to improve phenotypes obtained through random mutagenesis and/or *in vivo* evolution [41]. Applications of WGS for improving butanol tolerance have recently been reported [51–52]. *C. acetobutylicum* DSM 1731 mutants able to tolerate up to 19 g/l butanol were isolated, although butanol titers obtained by batch fermentations using this strain did not exceed 15.3 g/l [52].

The same approaches could be successfully applied for developing other ethanol tolerant or butanol resistant cellulolytic strains.

### Engineering efficient ethanol production in cellulolytic microorganisms

Several cellulolytic bacteria, such as *Clostridium sp*. (*e.g*. *C. thermocellum* and *C. cellulolyticum*), and fungi, such as *Rhizopus*, *Aspergillus*, *Neocallimastix*, and *Trichoderma*, can synthesize ethanol although their yields and/or titers and /or productivities are insufficient for direct utilization in CBP [6–7, 14]. Ethanol can be produced from pyruvate via two pathways (Figure 2): (i) pyruvate oxidative decarboxylation by Pfor and subsequent acetyl-CoA reduction to acetaldehyde and finally to ethanol; (ii) the pyruvate decarboxylase (Pdc) catalyzed conversion of pyruvate to acetaldehyde which is reduced by alcohol dehydrogenase (Adh) (Fig. 2). Clostridia generally employ the first pathway, however, a Pdc gene has been identified on the pSOL1 megaplasmid of *C. acetobutylicum* [36].

Rational metabolic engineering to increase ethanol yield and purity has been performed by two main strategies: introduce heterologous gene, and disrupt genes involved in by-product formation that compete with ethanol synthesis [34].

The first strategy, by employing the expression of *Zymomonas mobilis* Pdc and Adh genes, was applied to *C. cellulolyticum* [53]. Yet, acetate was the main end-product of the recombinant *C. cellulolyticum*. As compared to the wild type strain, acetate production was improved by 93% whereas final ethanol concentration was increased by 53% only [53].

Significant ethanol yield improvement was recently obtained by targeted gene disruption. The inactivation of a single gene, i.e. *hdb*, involved in butyrate synthesis in *C. acetobutylicum*, led to a strain with impressive ethanol yield (i.e. 0.38 g/g of glucose) and productivity (i.e. 0.5 g/l/h) [36]. Fed-batch cultures of the engineered *C. acetobutylicum* resulted in final ethanol amount of 33 g/l [36]. Ethanol titer obtained by a *hdb*-deficient *C. butyricum* was 18-fold higher that in the WT strain [35]. *hdb* deletion could be applied for improving ethanol production in butyrate producing cellulolytic clostridia, such as *C. cellulovorans* and *C. thermopapyrolyticum* [54].

Repression of acetate biosynthesis by inactivation of Pta and/or Ack has been suggested as a key modification for driving pyruvate flux towards ethanol [16]. However, disruption of the *pta* gene in *C. thermocellum* did not increase final ethanol amounts and led to severe growth deficiency as concerns both growth rate and final biomass [16]. Actually, acetyl-CoA conversion to acetate is a key pathway for metabolic energy production via SLP in clostridia [7, 35]. Indeed, attempts to construct *pta* or *ack* inactivated *C. cellulolyticum* strains were unsuccessful, so far [34].

Strategies employing Ldh disruption were more successful. A *C. cellulolyticum* H10 double mutant, where both Ldh paralogs, i.e. Ccel_2485 and Ccel_0137, were disrupted showed remarkable production of 0.27 g of ethanol per g of crystalline cellulose [34]. Similar ethanol yields from crystalline cellulose were obtained with a *C. thermocellum* strain that was constructed by both *ldh* and *pta* disruption [32]. However, maximum reported titers (5.61 g/l) remain low for this strain application to CBP [32].

Impressive results were obtained by deletion of *ack*, *ldh* and *pta* in the hemicellulolytic *T. saccharolyticum* [30]. The engineered strained showed homoethanologenic metabolism with maximum ethanol productivity and titer up to 2.2 g/l/h and 65 g/l, respectively [6, 30].

### Alternative strategies for engineering butanol production in cellulolytic strains

All natural butanol producers belong to the genus *Clostridium*. The highest butanol amounts are synthesized by *C. acetobutylicum*, *C. beijerinckii*, *C. saccharobutylicum*, and *C. saccharoperbutylacetonicum* [39]. Development of *C. acetobutylicum* or *C. beijerinckii* strains with improved butanol production (i.e. titers up to 19 g/l) by random mutagenesis or rational metabolic engineering was reported [41, 45]. However, none of these strains can directly ferment cellulose. Few cellulolytic clostridia producing very low butanol amounts include four recently isolated strains [54–55]. By developing effective gene manipulation tools, butanol production in these microorganisms could be improved by applying strategies that were previously set up in more established butanol producers.

The expression of the clostridial butanol biosynthetic pathway in heterologous hosts, such as native cellulolytic bacteria, seems an alternative promising strategy. The whole *C. acetobutylicum* butanol pathway, i.e. *thl*, *crt*, *bcd*, *etfB*, *etfA*, *bcd*, and *adhE* genes (Figure 2), was introduced in well-established and/or solvent tolerant heterologous hosts (e.g. *E. coli* and *Pseudomonas putida*), but the highest reported butanol titers, i.e. by *E. coli* BUT2, were 1184 mg/l [56–57]. Inefficient or imbalanced heterologous gene expression and low catalytic efficiency of some *C. acetobutilicum* enzymes (i.e. thiolase and butyryl-CoA dehydrogenase), have been hypothesized as the main causes of such low butanol production [57–58]. A chimeric butanol biosynthetic pathway was constructed in *E. coli* by assembling genes from three different organisms [58]. The clostridial Bcd was replaced by *Treponema denticola trans*-enoyl-CoA reductase (Ter). Both enzymes catalyze crotonyl-CoA reduction to butyryl-CoA, but Ter reaction is more exoergonic since it does not involve concomitant Fd reduction. Anaerobic fed-batch cultures of recombinant *E. coli* resulted in the impressive production of 15 g/L of butanol [58].

Strategies for efficient expression of the *C. acetobutylicum* butanol biosynthetic pathway in other *clostridium sp*. hosts, such as *C. tyrobutyricum*, might be less complex [59]. Since *C. tyrobutyricum* possesses its own butyrate biosynthetic pathway, the introduction of the *C. acetobutylicum* acetaldehyde/alcohol dehydrogenase AdhE2 and either *ptb* or *ack* inactivation, significantly diverted carbon flux from acetate and butyrate to butanol. About 10 g/l butanol was obtained by glucose fermentation [59]. This strategy could be successfully applied to other butyrate producers, such as cellulolytic *C. cellulovorans*.

**Figure 3:**
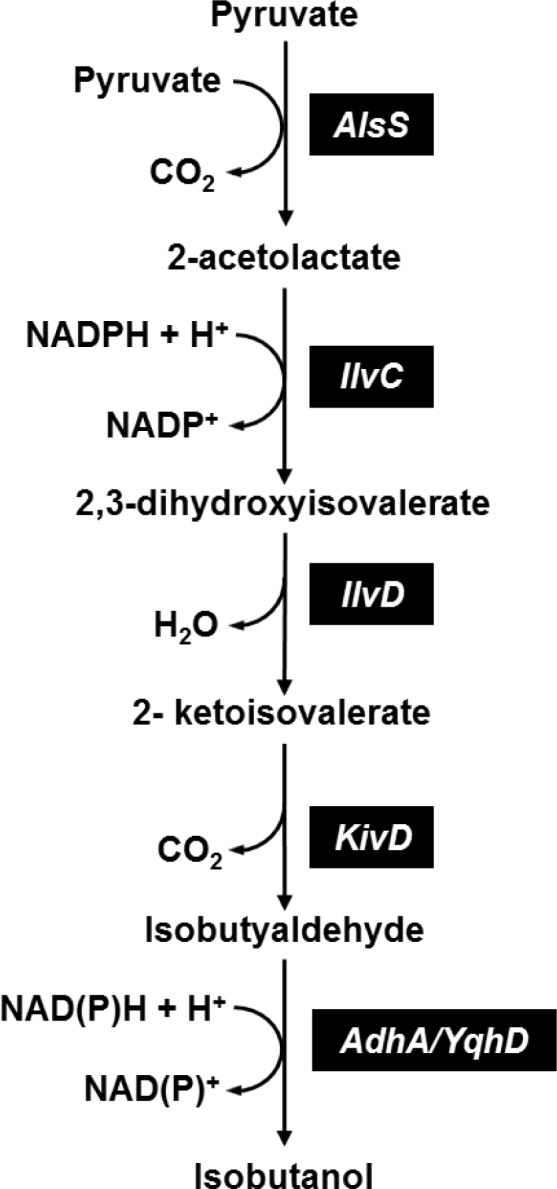
**Synthetic pathway for isobutanol production in *C. cellulolyticum* [15]**. Abbreviations: AlsS, *B. subtilis* a-acetolactate synthase; IlvC, *E. coli* acetohydroxyacid isomeroreductase; IlvD, *E. coli* dihydroxy acid dehydratase; KivD, *L. lactis* ketoacid decarboxylase; AdhA/YqhD *E. coli* and *L. lactis* alcohol dehydrogenases.

For a long time only the clostridial route to butanol synthesis has been known, but recently the *in vivo* construction of alternative pathways has been reported [19, 39]. Direct conversion of crystalline cellulose to isobutanol was performed by a modified *C. cellulolyticum* by the introduction of an engineered valine biosynthetic pathway [15] (Figure 3).

## Recombinant cellulolytic strategies: efficient heterologous cellulase system expression and beyond

The secretion of efficient designer cellulase systems in heterologous hosts is among the most challenging tasks of RCSs [60]. The strategies for both i) the design of efficient artificial cellulase systems and ii) their efficient secretion in strains with product formation features will be detailed in the next sections. In-depth analyses of natural cellulolytic microorganisms metabolism provide further insights for improving recombinant strains by central metabolic pathway engineering, as described in a further section.

### Design of efficient artificial cellulase systems: from nature to application

Minimal enzyme systems able to catalyze efficient cellulose hydrolysis contain at least an exoglucanase (Exg, i.e. either a cellodextrinase, EC 3.2.1.74, or a cellobiohydrolase, EC 3.2.1.91), an endoglucanase (Eng, EC 3.2.1.4) and a β-glucosidase (Bgl, EC 3.2.1.21) [3]. Yeast strains secreting a Bgl, a Exg and a Eng were able to directly ferment pretreated Whatman paper to ethanol with yields up to 94% of the theoretical maximum [61]. If RCSs involving heterologous expression of complexed cellulases (i.e. minicellulosomes or designer cellulosomes) are concerned, the additional expression of a scaffolding protein that consists of at least two cohesins is required for functional complex assembly [3].

In both free-cellulase and cellulosome biosynthesizing native organisms, optimal biomass degradation is obtained by secretion of non-equimolar ratios of different protein components with Exgs generally among the most abundant enzymatic subunits [3]. Indeed, a *Saccharomyces cerevisiae* strain that was engineered by introduction of two heterologous cellobiohydrolases only was able to hydrolyze up to 6 g/l of crystalline cellulose in 168 hours [62]. However, an in depth rationale able to explain and possibly predict which enzyme partners can act with the highest synergism degree is currently unavailable. Such information is essential to design optimized mixtures containing the minimal number of enzymatic subunits enabling efficient cellulose hydrolysis.

Exgs generally have a tunnel-shaped active site which retains a single glucan chain and prevents it from re-adhering to the cellulose crystal, thus enabling them to catalyze processive crystalline cellulose degradation from either the reducing or non-reducing end [9, 63]. Engs instead have cleft-shaped open active site which can cleave internal bonds of amorphous cellulose only [9, 63]. However, processive Engs, which are active on crystalline cellulose also, have also been discovered. Most processive Engs consist of a family 9 catalytic domain attached to a family 3c CBM [9, 649]. CBMs promote cellulase stable binding to cellulose, yet they allow the enzymes to diffuse along the cellulose chain. In some cases, CBM ability to disrupt non-covalent interactions between cellulose chains of crystalline cellulose has been demonstrated [63, 65].

A number of experimental observations indicate that cellulosomes are more effective than free enzymes, with special regards to insoluble (i.e. crystalline) cellulose hydrolysis, likely because the improved proximity enhances enzyme synergism [10–12]. However, in-depth understanding of the mechanisms that drive protein assembly and spatial organization in such complexes is still incomplete [8–9]. Cellulosome-biosynthesizing microorganisms adapt complex composition to the available substrate(s) and assembly non-equimolar ratios of the different subunits for optimal substrate degradation [66–68]. Cellulosome composition likely depends on both the relative amounts of available subunits and their differential affinity for cohesin domains, but with different extents depending on the microbial strain [69]. While within *C. cellulolyticum* and *C. thermocellum* cohesin-dockerin interaction seems to be non-selective or characterized by slightly different dissociation constants, up to 100-fold differences in binding affinities have been observed in *C. josui* and *C. cellulovorans* [11, 69].

Recent studies showed that linker regions that connect cohesins in scaffoldins are crucial for cellulosome plasticity and catalytic efficiency [70–71]. Linker flexibility enables cellulosome conformation to adapt to the substrate and allows glycosyl hydrolases (GHs) to work in close synergism through proximity effect [70–71]. Linker flexibility and length appear key factors mainly for very complicated and cell-bound cellulosomes, that likely need more extensible conformations [71]. Recent studies suggest that CBM3s, apart from promoting cellulosome binding to the substrate, could also induce conformational changes in the quaternary structure of cellulosomes through direct interaction with linker segments [72]. Cohesin–dockerin dual binding mode, i.e. the ability of dockerin-containing proteins to bind the cognate cohesin by two different orientations, also contribute to complex plasticity [73].

Which catalytic efficiency on native substrates can be expected for minicellulosomes with respect to natural complexes? Experimental evidences suggest an almost linear correlation between the number of cohesins that are beared by a scaffoldin and the activity on crystalline cellulose [11, 74]. The specific activity on crystalline cellulose of *C. thermocellum* minicellulosomes assembled by three-cohesin bearing mini-scaffoldin CipA was about 38% as compared to complexes containing the full-length CipA, which consists of 9 cohesins [11]. However, specific activity of designer cellulosomes or cellulase mixtures can be significantly increased by enzymatic components with superior activity either selected among the continuously increasing number of newly isolated GHs or developed through directed evolution or rational design [9, 12].

### Strain engineering with designer cellulase systems

Cellulolytic aerobic fungi (e.g., *Trichoderma reesei*) usually secrete high amounts (i.e. >1 to 10 g/l) of GHs. Although cellulosome-forming microorganisms biosynthesize lower cellulase levels (i.e. 0.1 g/l), cellulase amount as high as 10 to 20% (w/w) of whole cellular proteins was estimated in *C. thermocellum* [12].

State-of-the-art molecular biology enables the production of large amounts of cellulases in heterologous hosts by choosing one among the several mechanisms, at either mRNA or protein level, that regulate gene expression in microorganisms [60, 62].

Heterologous cellulase gene expression under the control of constitutive transcriptional promoters appears the most appropriate for strains aimed to biorefineries, since it avoids the non negligeable supplemental cost of large amounts of specific inducers [60]. By randomized or combinatorial methods, libraries of transcriptional promoters showing strengths within a range of three orders of magnitude can be easily constructed [75]. Improvement of mRNA stability and translation efficiency can be used as further tools to increase the expression of heterologous cellulases (for extensive review refer to [60])

A more challenging task of RCSs is the coordinated expression of multiple heterologous genes that are required for efficient cellulose degradation [60, 76]. Since cellulase system optimal activity is obtained for non-equimolar ratios of the different components, the use of multiple transcriptional units under different promoter control will probably be required. As detailed understanding of mRNA processing and post-trascriptional mechanisms in microorganisms is increasing, more elegant systems, e.g. fine tuning of artificial polycistronic operons by differential RNA stability and/or translation efficiency in bacteria, will be probably available. The design of artificial multifunctional GHs and/or “covalent” cellulosomes could provide efficient cellulose hydrolysis without the need of coordinated multiple gene expression [77]. The engineering of cellulases with superior activity on native substrates could also compensate for low secretion yields.

The main concern of RCSs is to find efficient and reliable secretion methods. The products of genes coding for clostridial cellulosomal components including their original signal peptide, were efficiently secreted by *C. acetobutylicum* and *Lactobacillus plantarum* [60, 78]. In other cases, efficient cellulase secretion was promoted by the replacement of original signal peptides by either signal peptides of efficiently secreted autologous proteins or optimized synthetic sequences [79–80]. Nowadays, insufficient understanding of high complexity and specificities among different microorganisms in protein secretion mechanisms, severely limits the number of targeted approaches that can be used for improving heterologous protein secretion [60]. We are currently unable to predict if a cellulase will be secreted in high amounts in a recipient strain or it will result in saturation of membrane translocation mechanisms and cell toxicity. Nonetheless, significant progress has been achieved by trial and error approaches, as well documented by studies on *C. acetobutylicum* and *S. cerevisiae* [62, 78, 81]. First attempts to express *C. cellulolyticum* Cel48F and Cel9G in *C. acetobutylicum* were unsuccessful. *C. acetobutylicum* deficiency of specific chaperone(s) that maintain family 48 and 9 GHs in a competent state for translocation was hypothesized [78]. However, Cel48F/Cel9G engineering with CBM3a and X2 modules of the *C. cellulolyticum* CipC scaffoldin prevented toxic effects and triggered enzyme secretion in *C. acetobutylicum* [81]. Prior to this study, the function of X2 domains was unknown. By selecting the most efficiently secreted enzymes from a large panel of heterologous Exgs, recombinant *S. cerevisiae* secreting up 1 g/l of cellobiohydrolases could be engineered [62]. Secreted heterologous cellulase amount was estimated as high as 4% of total cell protein of the recombinant *S. cerevisiae*, demonstrating that with sufficient efforts secretion of cellulase levels which are comparable to those observed in native cellulolytic strains is possible. Selected strains from *Kluyveromyces spp*. and *S. cerevisiae* expressing a library of cellulases were able to directly convert crystalline cellulose up to 0.4-0.5 g/l of ethanol without any externally added enzyme [82]. Furthermore, cultures of such engineered strain were able to ferment crystalline cellulose to ethanol with 30% of the maximum theoretical yield, when supplemented with commercial β-glucosidase [62].

In order to avoid the hydrolysis of the heterologously expressed cellulases, utilization of protease inactivated strains, such as *B. subtilis* WB800 and *L. lactis* HtrA mutants, may be required [79, 83].

Microbial cell surface binding enhances cellulase activity [12, 79]. Higher activity of cell-bound as respect to cell-free cellulosomes is obtained by limited escape of hydrolysis products and minimal distance products must diffuse before the cellular uptake occurs [12, 79]. The effect of such improved synergism is particularly evident on crystalline cellulose as compared with amorphous substrate degradation [12].

So far, designer cellulosomes binding up to 3 catalytic subunits have been functionally displayed on the surface of engineered microbial hosts. Such minicellulosomes have covalently been linked to the cell wall of the yeast *S. cerevisae* by means of agglutinin/flocculin display system [68, 84]. Trifunctional-minicellulosome-displaying *S. cerevisiae* was able to ferment amorphous cellulose to ethanol with 62% of the theoretical yield [84]. A non-covalent surface display system for lactic acid bacteria has been developed by target protein fusion with the C-terminal cA peptidoglycan binding domain of the major autolysin AcmA from *L. lactis* [60]. Fragments of the scaffolding protein CipA of *C. thermocellum* have covalently been anchored at the surface of *L. lactis* by fusing them with the C-terminal anchor motif of the streptococcal M6 protein, a sortase substrate [79]. A similar strategy was used to covalently link engineered *C. thermocellum* scaffoldins and cellulases to the *B. subtilis* cell wall [83]. Higher amounts of surface displayed constructs, i.e. about 3 x 10^5^ per cell, were estimated in engineered *B. subtilis* [83]. Recently, a designer cellulosome consisting of two scaffoldins, one involved in catalytic component binding and the other mediating cell-surface anchoring, was expressed in *S. cerevisiae* to improve complex-display level [85]. The recombinant strain was able to directly ferment crystalline cellulose to ethanol. Although the reported yields are low, as far as I know this is the first microbial strain able to biosynthesize by itself functional minicellulosomes enabling significant crystalline cellulose hydrolysis.

### Lessons from natural cellulolytic microorganisms

Research on native cellulolytic strains, suggests that cellulose hydrolysis is not the only bottleneck of cellulose metabolism [18]. As compared with soluble sugar metabolizing bacteria, anaerobic cellulolytic bacteria have limited carbon consumption rates and growth capabilities, which raise some concerns about maximum productivity that could be obtained by industrial cellulose bioprocessing.

However, improved conversion efficiencies by recombinant microorganisms developed by RCSs, could be obtained by introducing cellodextrin membrane transporters [86]. The uptake of cellulose hydrolysis product by cellulolytic microorganisms mainly consists in the transport of cellodextrins with a polymerization degree up to 7 which are degraded into the cytoplasm by phosphorolytic cleavage. Both cellodextrin uptake and phosphorolytic cleavage contribute to high bioenergetic benefits of cellulose with respect to glucose or cellobiose metabolism in native organisms [18]. These activities could be engineered in heterologous hosts for optimized valuable product yields and productivity by CBP.

Metabolic flux analysis could be an essential tool to identifying further bottlenecks of cellulose catabolism in native cellulolytic microorganisms and improve recombinant strains by rational engineering of central metabolic pathways. Alternatively, evolutionary engineering strategies by continuous culture under selective pressure could be applied to optimize cellulose overall metabolism in recombinant microorganisms [60].

## Conclusions and future perspectives

Both native recombinant strategies and recombinant cellulolytic strategies have made considerable progress. Outstanding results include the construction of *C. cellulolyticum* and *C. thermocellum* strains able to ferment crystalline cellulose to ethanol with yields close to 60% of the theoretical maximum and free cellulase-secreting or minicellulosome-displaying yeasts able to directly convert crystalline cellulose to ethanol [32, 34, 82, 85]. Yet, such strains are far to meet the yields, titers and productivities that are required for economically sustainable cellulose CBPs.

Rational engineering of biological systems so as to reach the high performances that are demanded by industrial processes will probably require the use of computational methods which can integrate: gene network regulation data; detailed information on *in vivo* enzyme catalytic parameters and metabolic fluxes; bioenergetics parameters (e.g. the energy demand of solvent tolerance mechanisms or cellulase biosynthesis, and biological reaction thermodynamics). Furthermore, this information will enable synthetic biology strategies to design new metabolic pathways for the conversion of cellulosic biomass into a virtually unlimited number of valuable products [39, 67].
